# Illuminating the genome: emerging approaches in CRISPR-Cas live-cell imaging

**DOI:** 10.1093/nar/gkaf1540

**Published:** 2026-01-15

**Authors:** Zhiguang Xiao, Yujie Sun

**Affiliations:** National Biomedical Imaging Center, College of Future Technology, Peking University, Beijing 100871, China; School of Life Sciences, State Key Laboratory of Membrane Biology, Biomedical Pioneering Innovation Center (BIOPIC), Peking University, Beijing 100871, China; National Biomedical Imaging Center, College of Future Technology, Peking University, Beijing 100871, China; School of Life Sciences, State Key Laboratory of Membrane Biology, Biomedical Pioneering Innovation Center (BIOPIC), Peking University, Beijing 100871, China

## Abstract

CRISPR-Cas-based live-cell imaging has rapidly become a central technology for studying genome dynamics with high specificity and flexibility. By coupling nuclease-deactivated Cas (dCas) with programmable guide RNAs, genomic loci can be tracked in living cells, providing direct insights into nuclear organization and chromatin behavior. While repetitive regions such as telomeres and centromeres are readily visualized, labeling non-repetitive loci remains more challenging due to weak signals and high background. Recent advances, including multicolor labeling strategies, innovative amplification systems based on dCas9 and single-guide RNA (sgRNA) engineering, and integration with novel fluorescent reporters, have markedly expanded the applicability of CRISPR imaging across the genome. These developments have expanded the multiplexing capacity of CRISPR imaging, improved signal-to-background ratios, and even enabled the visualization of non-repetitive genomic loci. Nonetheless, key challenges remain, including cellular toxicity, replication stress, and genomic instability associated with prolonged CRISPR expression. In this review, we summarize recent advances in CRISPR live-cell imaging and highlight key design trade-offs and biological constraints.

## Graphical Abstract

**Graphical Abstract:**
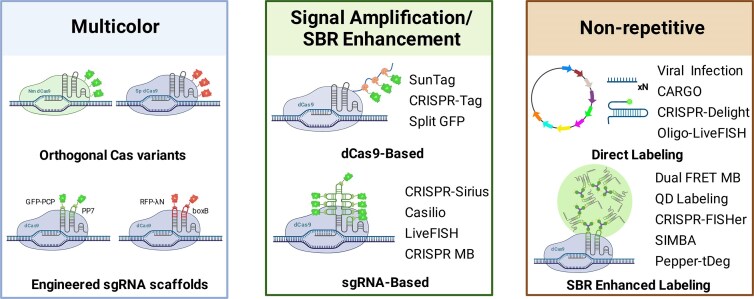

## Introduction

Understanding how the genome is spatially organized in three dimensions inside the nucleus, and how this organization evolves over time in living cells, is central to linking chromatin architecture with gene regulation, cell fate transitions, and disease progression [1–3]. Importantly, chromatin is not static: locus mobility, domain reorganization, and long-range contacts can change over minutes to hours, shaping transcriptional regulation, DNA replication and repair, and genome stability [4, 5]. Traditional approaches like chromosome conformation capture (Hi-C) and fluorescence *in situ* hybridization (FISH) have mapped genome architecture and spatial gene positioning, but these methods are typically performed in fixed samples (even though single-copy/low-copy genomic loci can be detected with modern DNA FISH variants) and therefore cannot capture real-time chromatin dynamics [6–9]. Prior to CRISPR-based imaging, live-cell labeling of defined genomic loci primarily relied on operator-array tagging systems (e.g. LacO/TetO knock-ins bound by fluorescent LacI/TetR; “FROS”) and engineered sequence-specific DNA-binding proteins such as zinc-finger proteins and transcription activator-like effectors [10–12]. However, these approaches typically require labor-intensive genome engineering (e.g. insertion of large operator arrays) or target-by-target protein design, can perturb local chromatin/transcription, and are challenging to scale for high-throughput multiplexed imaging [12–14]. Thus, there has been a strong impetus to develop reliable live-cell chromatin imaging techniques to observe genome behavior over time in intact, living cells. Such approaches provide critical insights into how 3D genome folding shapes chromatin compartments [15], guides cell-fate decisions [16, 17], drives developmental transitions [18], and is perturbed in disease states [19].

The adaptation of CRISPR-Cas technology for live-cell DNA imaging has transformed this field [20, 21]. The key breakthrough was the creation of a catalytically inactive Cas9 (dCas9) that can bind DNA targets without cutting them. Qi *et al.* first demonstrated such a “dead” Cas9 in 2013 as a programmable DNA-binding platform [22]. Shortly thereafter, Chen *et al.* fused dCas9 with green fluorescent protein (GFP) and showed that an RNA-guided dCas9 can tag specific genomic loci in living human cells [23]. In brief, a single guide RNA (sgRNA) and fluorescent dCas9 form an RNP complex that is guided to a complementary DNA sequence, enabling sequence-specific visualization of genomic loci in living cells. By simply designing an sgRNA to a sequence of interest, researchers could direct fluorescent dCas9 to that locus and track its position in real time [23]. This RNA-programmable targeting provides several key advantages over prior live-cell locus-labeling strategies: it avoids labor-intensive locus-specific protein engineering or large operator-array knock-ins and enables rapid retargeting to new endogenous loci by changing only the guide sequence. Early studies successfully labeled repetitive regions such as telomeres and centromeres, which contain many tandemly repeated target sites and thus yielded bright signals with only a few kinds of sgRNAs, and could also be extended to multicolor imaging through orthogonal Cas systems or engineered sgRNA scaffolds [23–28].

Labeling non-repetitive loci is intrinsically challenging because single-copy sites provide only one binding site per sgRNA-dCas9 complex, producing weak signals that are hard to distinguish from background [23, 29, 30]. To improve signal-to-background ratio (SBR)—defined here as the fluorescence contrast between the summed signal from locus-bound CRISPR complexes (signal; enriched foci at the targeted genomic site) and diffuse or nonspecifically accumulated fluorescence from unbound components (background; including nucleolar enrichment)—both the effector and the guide have been rationally re-engineered [4, 31–34].

Conceptually, SBR can be improved via two complementary routes: increasing on-target signal (e.g. higher fluorophore recruitment/brightness) and/or suppressing background (e.g. conditional/activatable fluorescence or reducing unbound reporter pools). These contrast-enhancement strategies can enable robust visualization of non-repetitive loci with only a few CRISPR complexes [35–38]. By contrast, alternative approaches achieve detectability by densely tiling dozens of sgRNAs across the target region [39–43], trading experimental simplicity for delivery burden and increased off-target risks.

Despite these successes, CRISPR-based imaging is not without drawbacks. Prolonged expression of dCas9 and sgRNAs can impose obstacles on replication or transcription [44–46], trigger DNA damage responses [47], and perturb chromatin states [48]. These effects underscore the need for careful dosage control and for engineering more transient or inducible delivery systems to minimize toxicity [48]. Importantly, many of these perturbations remain poorly quantified or inconsistently reported. CRISPR-based genome imaging has rapidly advanced from proof-of-concept to a versatile tool for tracking chromatin dynamics [49, 50]. Here, we synthesize current CRISPR imaging strategies with an explicit focus on engineering trade-offs and cellular constraints, aiming to guide more informed, balanced, and biologically safe application of these tools.

## Expanding the CRISPR toolbox for multicolor imaging

Achieving multicolor, multiplexed genome imaging hinges on assigning distinguishable fluorescent signatures to distinct DNA targets in the same cell. Two complementary routes are most effective: orthogonal Cas effectors [24, 25] and sgRNA scaffolds that recruit color-coded reporters [26, 27], which together enable simultaneous tracking of multiple loci and quantitative analyses of spatial relationships and chromosomal dynamics. Here, we focus on encoding principles for multiplexing; strategies that primarily increase imaging contrast (signal boosting and background suppression) are discussed in the next section, and additional constraints specific to unique/low-copy (non-repetitive) loci are discussed in the “Non-repetitive sequences imaging” section.

### Orthogonal Cas variants

**Figure 1.**
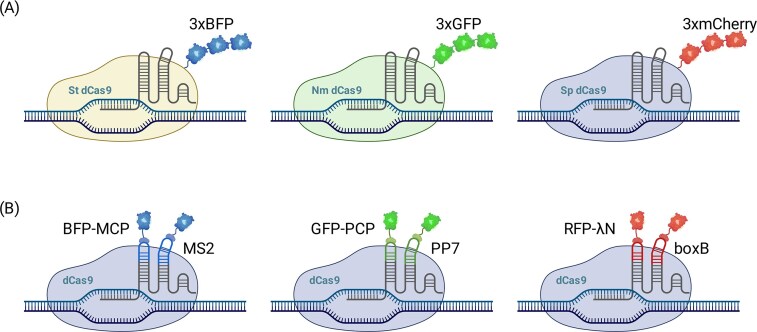
Strategies for multicolor CRISPR imaging. (**A**) Orthogonal Cas variants, *Streptococcus pyogenes* dCas9, *Neisseria meningitidis* dCas9, and *Streptococcus thermophilus* dCas9, are used to label distinct genomic loci with different colors in living cells. Each variant has its own unique PAM sequence and sgRNA recognition, allowing independent operation without cross-reactivity. (**B**) Engineered sgRNA scaffolds, such as MS2, PP7, and BoxB, are inserted into the sgRNA loops to recruit their matching coat proteins (MCP, PCP, and λN), which are fused to different fluorescent proteins for multicolor labeling. This allows multicolor visualization of multiple genomic loci using a single dCas9 protein.

A direct way to separate colors is to run multiple CRISPR systems in parallel, each with its own protospacer adjacent motif (PAM) logic, sgRNA, and fluorescent fusion so they operate independently without cross-reactivity. Here, “PAM logic” refers to the PAM sequence requirements of each Cas ortholog, which dictate targetability and underpin orthogonality between Cas variants. Early demonstrations combined *Streptococcus pyogenes* dCas9 and *Staphylococcus aureus* dCas9 to label two loci in different colors within single human cells [24, 25]. The concept was extended to three colors using dCas9 orthologs from *S. pyogenes, Neisseria meningitidis*, and *Streptococcus thermophilus*, each illuminating a distinct chromosomal site (Fig. 1A) [25]. This strategy scales with the availability of well-behaved orthologs and spectral channels, and it can be paired with Type V systems (e.g. Cas12a) for arrayed guide processing [41, 51]. Hybrids that combine DNA-targeting dCas9 with RNA-targeting dCas13 further enable concurrent visualization of a genomic locus and its transcript [52, 53], illustrating the versatility of orthogonal CRISPR mixes. Yet the practical ceiling of this approach remains unclear: only a handful of orthologs have been thoroughly benchmarked, PAM and sequence-context constraints restrict target choice, and the cumulative expression burden for two to three large effectors already challenges many primary or non-dividing cells.

### Engineered sgRNA scaffolds

An alternative is to keep a single dCas9 species and encode color information in the guide. In CRISPRainbow [26], orthogonal RNA aptamers such as MS2, PP7, and BoxB are inserted into the sgRNA loops to recruit their matching coat proteins (MCP, PCP, and λN) fused to distinct fluorophores (Fig. 1B). By assigning different aptamer combinations to different guides, six to seven visually separable labels were realized in individual cells using one dCas9 [26]. This design simplifies protein delivery and connects to a broader set of sgRNA scaffold strategies, where repeated protein-binding motifs not only enable multicolor labeling but also amplify signals by recruiting multiple fluorescent proteins, though at the cost of higher background. In practice, however, long scaffold inserts and multimeric coat-protein recruitment can perturb sgRNA folding and on-target binding, increase nonspecific nuclear accumulation (especially in the nucleolus), and demand finely tuned expression stoichiometry [27, 54–56]. These issues raise a critical question for future applications: how many colors and loci can realistically be tracked before background, mislocalization, and guide dropout outweigh the benefits of multiplexing?

Second-generation designs focused on improving stability, brightness, and multicolor flexibility. CRISPR-Sirius optimized sgRNA scaffolds (e.g. 8× MS2/PP7 with diversified linkers) to yield stronger signals and enable versatile multicolor labeling [33]. Shao *et al.* demonstrated sustained dual-color tracking of two loci using modified sgRNAs carrying MS2 and PP7 motifs, underscoring the potential of scaffold engineering for long-term imaging [27]. Casilio further expanded the toolbox by replacing phage coat proteins with programmable PUF domains that bind engineered RNA motifs, recruiting distinct fluorophores for multi-color, multi-locus imaging with a single dCas9 [35, 57, 58].

Together, orthogonal Cas systems and engineered sgRNA scaffolds have established a versatile foundation for multicolor CRISPR imaging, greatly enhancing the capacity to dissect spatial genome organization in living cells.

## Signal amplification strategies in CRISPR-dCas9 imaging

Fluorescent tagging of dCas9 enables direct visualization of genomic loci in living cells, but single fusion proteins only yield weak signals, particularly at low-copy targets. This section summarizes cross-cutting SBR-improvement strategies (signal boosting versus background suppression) that apply across target types. To address this limitation, diverse signal amplification strategies have been developed. These approaches fall into two main groups: (i) protein-based amplification through dCas9 engineering and (ii) RNA-based amplification by modifying the sgRNA.

### dCas9-based signal amplification

**Figure 2.**
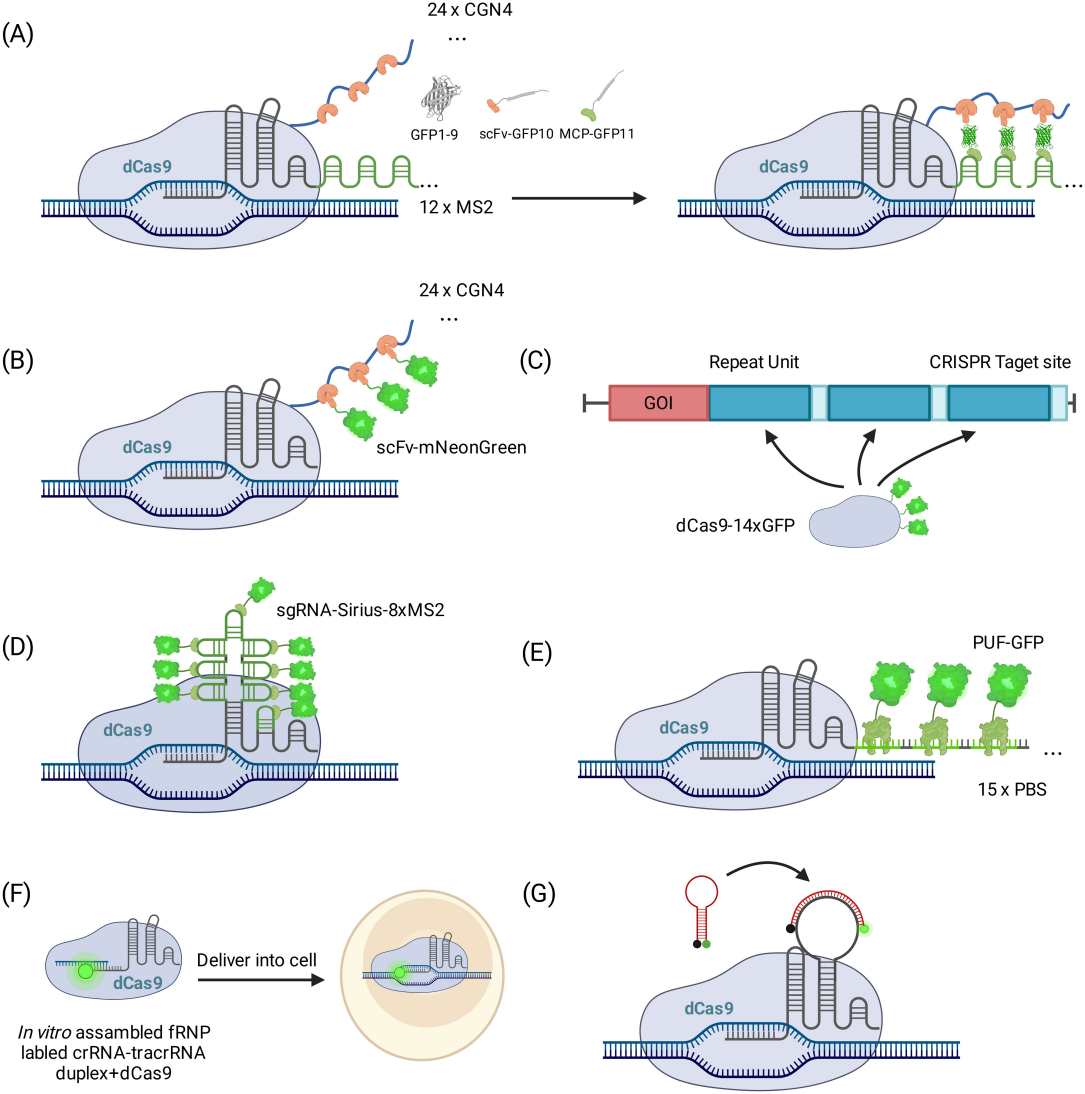
Signal amplification strategies in CRISPR-dCas9 imaging. (**A**) Split GFP: one binds to dCas9 via SunTag, another interacts with the sgRNA, and the third is freely expressed. The GFP fragments reassemble into a functional fluorophore only when CRISPR complexes co-localize at target sites, reducing background. (**B**) SunTag system, where dCas9 is fused with up to 24 tandem GCN4 peptide epitopes, each recruiting a single-chain variable fragment (scFv) tagged with a fluorescent protein. This strategy enhances signal strength, exemplified by the use of mNeonGreen. (**C**) CRISPR-Tag, where tandem CRISPR target sites are inserted near the locus of interest, and multiple sgRNAs recruit dCas9 clusters. (**D**) CRISPR-Sirius, where up to 8 aptamers are inserted into the sgRNA scaffold, significantly increasing brightness and enabling detection of low-copy loci. (**E**) Casilio system, where engineered sgRNAs containing multiple Pumilio recognition sites (PBS) at 3′ of sgRNA recruit fluorescent PUF proteins for signal amplification. This system allows multiplexed imaging of distinct loci with enhanced signal intensity. (**F**) LiveFISH, where fluorescently labeled crRNA:tracrRNA duplexes (gRNAs) are pre-assembled with dCas9 into ribonucleoprotein complexes for efficient delivery into cells, enabling fast and bright labeling of genomic loci. (**G**) CRISPR MB uses a fluorophore-quencher hairpin structure that de-quenches upon target engagement, enhancing SNR.

A straightforward strategy is to fuse multiple fluorescent proteins (FPs) to each dCas9. Ma *et al.* attached three tandem FPs (e.g. 3× GFP, 3× mCherry, and 3× BFP) to different dCas9 orthologs, enabling signal amplification of telomeres and distance measurements between chromosomal sites in single cells (Fig. 1A) [25]. To suppress background from dispersed fluorophores, later work introduced split fluorescent proteins, where GFP fragments reassemble into a functional fluorophore only when CRISPR complexes co-localize at target sites. In Chaudhary *et al.*’s work, GFP was split into three parts: one fragment associates with the scFv on dCas9 through the SunTag, another interacts with the sgRNA via MCP and the MS2 system, while the third fragment is expressed freely in the cell. (Fig. 2A) [59, 60]. When all three fragments met on target, the SBR increased by about six-fold compared with a regular SunTag-GFP setup, making the foci clearer and reducing off-target fluorescence.

More elaborate protein scaffolds have since been introduced. The SunTag system fuses up to 24 tandem GCN4 peptide epitopes to dCas9, each recruiting an scFv-FP [61]. This yields ∼20-fold stronger signals and allowed real-time imaging of human telomeres [61]. Ye *et al.* further optimized SunTag with mNeonGreen, achieving higher brightness and improved signal-to-background (Fig. 2B) [32]. Building on this design, an optogenetic SunTag system incorporating the light-inducible nuclear export tag (LEXY) selectively exports unbound fluorescent proteins under blue light, improving SBR by ∼2-2.5-fold [62].

Another elegant design is CRISPR-Tag, in which a short DNA cassette containing tandem CRISPR target sites is inserted near a locus of interest. Multiple sgRNAs recruit clusters of dCas9, and the signal is further boosted by split GFP complementation, with each dCas9 carrying up to 14 GFP fragments (Fig. 2C) [63]. Building on this, TriTag used a more compact DNA tag to enhance SBR and enabled simultaneous imaging of DNA, nascent RNA, and protein products, linking chromatin structure and transcriptional activity [64].

These protein-centric approaches provide strong and stable amplification, making them valuable for long-term imaging in robust immortalized cell lines where high expression levels are tolerable. However, they typically rely on sizable protein fusions (e.g. SunTag, 3× FP) or locus engineering (CRISPR-Tag/TriTag), raising barriers for delivery and genome editing in primary cells and whole organisms and increasing the risk of perturbing native chromatin context. In our view, these design constraints currently make most protein-centric amplification schemes difficult to deploy in primary or *in vivo* systems, highlighting an urgent need for next-generation “low-burden” amplification strategies that can decouple high SBR from heavy protein overexpression or genomic insertion.

### sgRNA-based signal amplification

Another strategy is to embed RNA aptamers within the sgRNA scaffold so that each dCas9-sgRNA complex recruits multiple fluorescent proteins. The improved CRISPR-Sirius design placed up to 8 aptamers in stable loops, significantly increasing brightness and enabling detection of low-repeat loci (Fig. 2D) [33].

The Casilio system employs engineered sgRNAs containing multiple PBS. Fluorescent PUF proteins then bind these motifs, amplifying signals. Because PUF-RNA recognition is programmable, Casilio can mix distinct PUF variants to multiplex loci. Cheng *et al.* showed that sgRNAs with 20-25 PBS repeats could label telomeres and centromeres simultaneously (Fig. 2E) [58]. To simplify delivery, Zhang and Song created Aio-Casilio, packaging all components into a single vector, improving efficiency and applicability [57]. Subsequent Casilio variants have improved orthogonality, introduced inducible clustering modules, and enhanced specificity, and in optimized formats even allowed single non-repetitive loci to be labeled with one sgRNA [35].

Other RNA-based strategies use direct fluorophore labeling. LiveFISH pre-assembles fluorescently labeled sgRNAs with purified dCas9 into ribonucleoprotein complexes, which are then delivered into cells by electroporation (Fig. 2F) [65]. This provides rapid and bright labeling without transcriptional delays and has been used in embryos and primary cells [65]. Chemically synthesized guides also permit multicolor stoichiometry control and reduced cellular burden, although the signals are transient as ribonucleoprotein complexes (RNPs) dilute over cell divisions. Similarly, CRISPR-molecular beacons hybridize fluorescent probes to engineered sgRNAs, using a fluorophore-quencher hairpin that de-quenches upon target engagement, lighting up only at target-bound complexes and thereby improving SBR (Fig. 2G) [66].

Despite advances, RNA-scaffold amplification remains constrained by bulky, repeat-rich sgRNAs, strict guide-binder stoichiometry, and vector size limits, which often hinder its scalability and application in complex experimental settings [4, 33, 35, 58]. Overly long sgRNAs can further depress U6-driven transcription, reducing the actual amount of functional guide in cells [33]. Increased expression burdens elevate background, RNPs are short-lived, and molecular beacons are sensitive to temperature and nucleases, which together limits durability and high-level multiplexing [42, 65–67]. Recent work showed that plasmid-based sgRNA delivery can produce backbone-derived transcripts that generate false-positive foci, particularly when sgRNAs carry probe-binding/aptamer sites; transfecting only sgRNA expression cassettes can substantially reduce such artifacts [68]. Moving forward, these limitations call for more compact, robust RNA scaffold designs with enhanced stability and reduced delivery burden [33]. One promising direction could be leveraging synthetic biology to create optimized, smaller guide architectures that minimize these challenges while maintaining signal integrity [69]. For example, scaffold modules and aptamer-binder pairs could be rationally redesigned or evolved to be more compact and orthogonal while preserving strong recruitment at bound loci [33, 35, 58]. In parallel, stabilizing guide RNAs through chemical/structural engineering and incorporating programmable, conditional RNA elements could reduce degradation and unbound reporter pools, thereby improving scalability and SBR for long-term and multiplex imaging [69, 70].

## Non-repetitive sequences imaging

Imaging non-repetitive genomic loci is substantially more challenging than repetitive regions (e.g. telomeres and centromeres) because far fewer target sites are available per locus, resulting in fewer DNA-bound complexes and consequently weaker fluorescence that can be comparable to diffuse background [4]. This challenge is rooted in genome architecture: repeat and repeat-derived DNA may account for >66%-69% of the human genome [71], whereas the protein-coding exome comprises only ∼1%-1.5% and is predominantly unique [72]; accordingly, these non-repetitive sequences constitute a central focus of functional and disease-oriented genomics. In addition, the same nominal number of sgRNAs is often not equivalent between repetitive and non-repetitive targets: repetitive arrays provide densely spaced binding sites that promote strong local clustering of dCas complexes within a diffraction-limited focus, whereas non-repetitive loci typically require tiling guides across a broader genomic window [63]. However, PAM availability and chromatin accessibility constrain usable sites, and dense tiling is further limited by target-site spacing and steric hindrance between neighboring dCas9 complexes, reducing achievable simultaneous occupancy [73]. Moreover, competition among multiple guides for a finite pool of dCas9 and heterogeneous guide loading/expression across cells may further lower effective on-target occupancy and fluorophore density. Accordingly, non-repetitive locus imaging typically requires strategies that increase the number or occupancy of locus-bound complexes, enhance per-complex brightness, and suppress background, while keeping delivery burden and toxicity manageable [74]. This section focuses on strategies tailored to unique/low-copy loci, where limited target sites impose additional constraints on occupancy, brightness, and background suppression.

### Direct labeling of non-repetitive sequences

**Figure 3.**
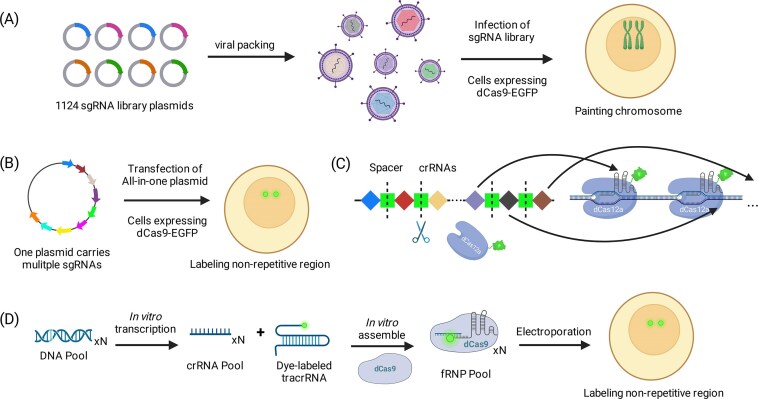
Strategies for direct labeling non-repetitive sequences in living cells. (**A**) Viral sgRNA library infection: 1124 sgRNAs are delivered via lentivirus to “paint” chromosome 9. This strategy successfully reveals conformational changes across the cell cycle. (**B**) CARGO, multiple sgRNAs are packaged into a single plasmid for simplified transfection and reproducible visualization of non-repetitive loci. (**C**) CRISPR-Delight, Cas12a arrays encode 6-10 sgRNAs per array, naturally processing tandem spacers into crRNAs in cells. This approach enables single-copy locus visualization with reduced plasmid burden. (**D**) Oligo-LiveFISH, a pool of fluorescent crRNAs, is assembled *in vitro* and delivered as pre-formed RNPs for live-cell imaging of non-repetitive genomic loci.

Early direct-labeling strategies addressed the intrinsically weak signal at non-repetitive loci by tiling multiple sgRNAs. For example, Chen *et al.* targeted the MUC4 locus with 73 unique sgRNAs via lentivirus and showed that as few as 36 were sufficient to produce a detectable fluorescent locus, enabling tracking of its dynamics, though with limited SBR [23]. On a larger scale, Zhou *et al.* delivered up to 1124 sgRNAs via lentivirus to “paint” chromosome 9 in HeLa cells, successfully revealing its conformational changes across the cell cycle (Fig. 3A) [75]. These early demonstrations proved feasibility, but they largely depended on large sgRNA pools and viral expression, creating a high delivery/engineering burden and cell-to-cell heterogeneity while increasing background from excess unbound complexes [23, 75]. As a result, performance and portability were often inconsistent across cell types, motivating strategies that boost SNR with fewer guides and more controlled, low-perturbation delivery.

To reduce the heavy delivery burden of dozens or hundreds of sgRNAs, researchers have turned to multi-sgRNA assembly strategies. Shao *et al.* cloned 20 sgRNAs into a single vector and visualized single-copy loci such as HER2 and MUC4 [40]. Similarly, CARGO uses a modular assembly system that packages 12 sgRNAs into one plasmid, simplifying transfection and enabling reproducible imaging of non-repetitive sites (Fig. 3B) [39].

A complementary approach leverages natural guide processing. CRISPR-delight exploits Cas12a arrays, which naturally process tandem spacers into multiple crRNAs in cells [41]. By encoding up to 48 guides in one array, sufficient binding can be achieved for single-copy locus visualization with dCas12a-GFP (Fig. 3C). Multiple arrays can be combined for multicolor or multi-locus labeling, reducing plasmid burden and simplifying delivery [41].

Another strategy is Oligo-LiveFISH [42], developed as an extension of the earlier work on Live-FISH [65]. In this method, a pool of over 12 kinds of fluorescent crRNAs is assembled *in vitro* and delivered as pre-formed RNPs, enabling efficient live-cell imaging of non-repetitive genomic loci without the need for stable cell line generation (Fig. 3D). More recently, Liu *et al.* developed PRO-LiveFISH [76], which leverages expanded genetic alphabet technology in which unnatural base pairs (UBPs, denoted as X-Y) function as an orthogonal third base pair to enable site-specific incorporation of fluorophore labels into sgRNAs. Notably, PRO-LiveFISH enables multiplexed imaging of up to six non-repetitive loci with as few as ∼10 sgRNAs per locus [76]. Together, these later approaches reduce reliance on large viral guide pools and improve practical deployability for unique/low-copy loci.

However, high guide counts and large assemblies inflate vector size and cloning complexity, increase recombination risk, and raise background from off-target or uneven guide expression. Cas12a arrays add PAM and sequence-context constraints. RNP-based methods are transient and can be costly to multiplex. Together these factors limit scalability for long tracking windows and high multiplexing. To overcome these issues, it may be crucial to focus on developing more efficient guide design strategies and optimizing delivery systems to balance high throughput with low toxicity.

### SBR enhanced labeling of non-repetitive sequences

**Figure 4.**
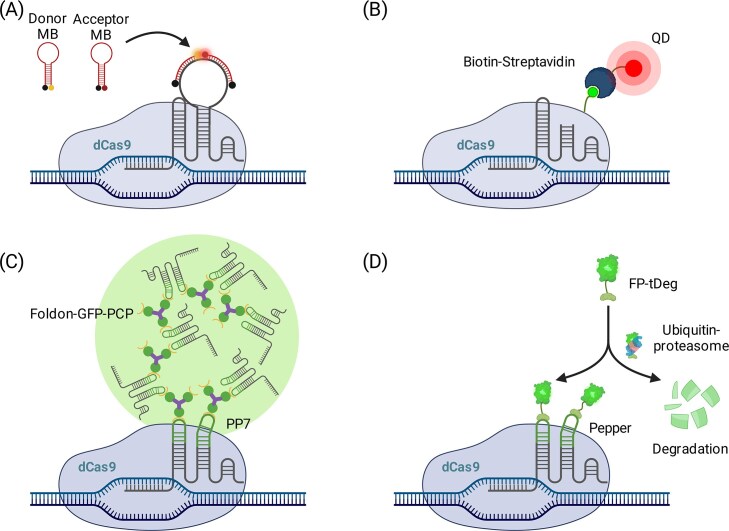
SBR enhanced labeling of non-repetitive sequences. (**A**) Dual FRET MB, the donor- and acceptor-labeled probes are designed to bind adjacently to the target sequence, producing FRET only when CRISPR complexes are bound at the target locus. This system enables single-copy locus detection with as few as three sgRNAs. (**B**) QD labeling, dCas9 is fused with biotin and conjugated with streptavidin-QD probes, allowing dual-color labeling of integrated HIV-1 DNA using just two sgRNAs. (**C**) CRISPR-FISHer, PP7 system recruits multiple PCP-GFPs, while the FUS low-complexity domain induces phase separation, concentrating the signal into bright foci. Allowing single-copy locus imaging and tracking rare events like double-strand breaks. (**D**) CRISPR/Pepper-tDeg, an sgRNA containing Pepper aptamers, recruits fluorescent proteins fused to a degron (tDeg) that is rapidly degraded unless bound. This method enables real-time single-copy imaging with minimal background.

Another direction is to improve the SBR so that only a few sgRNAs are needed. Similar to the CRISPR molecular beacon (CRISPR-MB) system (Fig. 2G) [66], Mao *et al.* extended this design to a dual FRET MB system, inserting two binding sites so that donor- and acceptor-labeled probes bind adjacently and produce FRET (Fig. 4A) [67]. Only target-bound complexes de-quench the hairpin and emit fluorescence. This enabled single-copy locus detection with only three sgRNAs, demonstrated at MUC1 and intergenic sites [67]. In practice, performance depends on probe Tm, spacer placement, and intracellular nuclease resistance, which together tune on-target brightness and background.

An alternative and innovative approach involves the use of quantum dots (QDs) [77]. Ma *et al.* successfully fused peptide tags to dCas9 and conjugated them with streptavidin-QD probes (Fig. 4B), using just two sgRNAs to image HIV proviral integration sites. This enabled the dual-color labeling of unique loci, with QDs providing intense, stable fluorescence signals that are ideal for detecting low-copy sequences. Due to their high quantum yield, quantum dots provide stronger and longer-lasting signals, which are crucial for imaging low-abundance targets such as integrated HIV-1 DNA.

RNA scaffold engineering also boosts signal. We have already discussed that the Casilio system embeds multiple programmable PUF aptamers into sgRNAs to recruit fluorescent proteins. In addition, sgRNAs that fail to assemble with dCas9 are comparatively unstable and are more readily degraded, which can reduce the pool of free scaffold available to recruit PUF-FPs and ultimately reduce the SBR. Building on previous work [57, 58], Clow *et al.* demonstrated that a single sgRNA with 15 binding sites could successfully visualize MUC4 alone (Fig. 2E) [35] when component stoichiometry is tuned to maintain a favorable SBR. They extended this to two loci in two colors and developed PISCES, in which three sgRNAs tagged adjacent non-repetitive segments in different colors, revealing local 3D structure. Improvements to MS2-based scaffolds have also helped. Qin *et al.* designed a 16× MS2 system, enabling detection of MUC4 with eight sgRNAs, and with further optimization, as few as four guides sufficed [4]. These scaffold strategies benefit from high recruitment stoichiometry but must preserve sgRNA folding and dCas9 binding while minimizing nucleolar accumulation, which likely arises when overly long, multimeric sgRNAs misfold and get trapped with surplus coat proteins in the nucleolus rather than being delivered to DNA targets.

More recently, condensate-based amplification has emerged. Lyu *et al.* introduced CRISPR-FISHer, adding a FUS low-complexity domain to dCas9-SunTag [36]. The SunTag recruits multiple PCP-GFP, and the FUS-IDR induces local phase separation, concentrating signal into bright foci (Fig. 4C). Similarly, Peng *et al.* created SIMBA, fusing HP1α to dCas9-SunTag [37]. HP1α condensates both amplify GFP signal and recruit SUV39H1/2 to deposit H3K9me3, coupling non-repetitive regions imaging with chromatin modulation [37].

Finally, CRISPR**/**Pepper**-**tDeg uses an sgRNA with Pepper RNA aptamers (Fig. 4D) [38, 78]. These recruit fluorescent proteins fused to a degron (tDeg) that causes rapid degradation unless bound. Because FP-tDeg is degraded unless stabilized by Pepper-sgRNA binding, the free reporter pool is depleted and fluorescence becomes strongly enriched at target-bound loci, yielding low background.- With one sgRNA, they achieved real-time single-copy imaging with minimal background and demonstrated two-color tracking [38].

Across these “few-guide” strategies, performance can be constrained by probe delivery and stability as in CRISPR-MB; by particle size and blinking in QD-based labeling, which also makes it harder to discriminate true on-target foci from transient bright spots or aggregates; by sgRNA folding and nucleolar accumulation in Casilio or MS2 designs; by potential chromatin perturbation in condensate-based systems such as CRISPR-FISHer or SIMBA; and by dependence on protein turnover for signal accumulation in Pepper-tDeg. In our view, the safety and robustness of these “few-guide” designs remain under-tested. A clear next step is to establish standardized benchmarks for toxicity, chromatin perturbation, and mislocalization rather than optimizing solely for imaging SBR.

## Overview of emerging strategies in CRISPR-Cas live-cell imaging

Overall, CRISPR-Cas imaging technologies have rapidly evolved into a versatile toolbox for interrogating genome organization in living cells. Orthogonal Cas variants and engineered sgRNA scaffolds have established the foundation for multicolor imaging, allowing simultaneous visualization of multiple loci and enabling quantitative studies of spatial genome relationships [24–27]. In parallel, both protein-centric and RNA-centric amplification systems have significantly improved the SBR, extended the detection window, and facilitated long-term live tracking with higher sensitivity [32, 33]. Furthermore, creative solutions for non-repetitive locus labeling—including tiled or multiplexed sgRNA strategies [39, 40], compact Cas12a arrays [41], fluorescent crRNA delivery [42], and condensate-driven signal amplification [36, 37]—have made it possible to visualize single-copy genomic regions that were once inaccessible.

Despite these advances, each strategy carries trade-offs in terms of complexity, sensitivity, perturbation, and applicability to different cell types or organisms. For example, SunTag-based amplification achieves strong and stable signals but requires extensive protein engineering [32], whereas Oligo-LiveFISH and PRO-LiveFISH enable rapid deployment without stable cell lines but are more transient in nature [42, 76]. Similarly, scaffold-based approaches offer flexibility in multicolor labeling but can increase background if over-saturated [26, 27].

To help readers quickly identify a suitable strategy, we recommend starting from four practical questions.

1. Is the target repetitive or non-repetitive? Repetitive loci (e.g. telomeres/centromeres) typically require only basic dCas9-FP labeling or simple multicolor implementations (orthogonal Cas variants/sgRNA scaffolds) because many binding sites naturally boost signal. Non-repetitive loci generally require either more bound complexes (tiled/multiplexed guides, compact Cas12a arrays) and/or higher per-complex brightness (protein/RNA recruitment scaffolds, condensate-assisted clustering, or fluorescent guide delivery).
2. Is fast deployment across cell types required? If stable cell line generation is impractical (e.g. primary/sensitive cells), prioritize transient approaches, especially pre-assembled RNP delivery with fluorescent guides (e.g. Oligo-LiveFISH/PRO-LiveFISH) or other short-pulse strategies. If engineered cell lines are feasible, stable expression with controlled titration can support longer observation windows and repeated experiments.
3. What is the dominant failure mode: weak signal or high background? If signal is limiting, choose signal-boosting designs (multimeric recruitment, scaffold-based recruitment, condensate-based clustering, brighter probes). If background is limiting, choose background-suppressing designs (conditional/activatable fluorescence or strategies that reduce unbound reporter pools).
4. Is the goal multi-locus/multicolor imaging or long-duration single-locus tracking? For multiplexing, orthogonal Cas systems and scaffold encoding scale most readily; for long-duration tracking, prioritize designs with stable labeling and manageable photobleaching/phototoxicity, even if multiplexing capacity is reduced.

To provide a concise overview of these diverse methods, we have compiled a comparative table that outlines representative CRISPR imaging strategies and their primary application scenarios, along with their major advantages and limitations (Table 1). We hope this reference will serve as a practical guide for researchers to select the most suitable approach according to their experimental needs.

**Table 1.** Representative CRISPR-Cas live-cell genome imaging strategies, application scenarios, and key trade-offs

Method	Imaging sequence	Principle	Key refs	Advantages	Limitations
Direct dCas9-FP	Repetitive	Fluorescently tagged dCas9 is guided by sgRNA to genomic DNA	[23]	Minimal components; rapid retargeting by sgRNA design; Milestone	Low SBR; diffuse nuclear/nucleolar background
Orthogonal Cas variants	Repetitive	Multiple dCas9 orthologs with distinct fluorescent tags	[24, 25] (Fig. 1A)	Enable simultaneous multi-locus imaging with minimal cross-talk	Limited by availability of well-behaved orthologs
CRISPRainbow/sgRNA aptamer scaffolds	Repetitive	Insert RNA aptamers (MS2, PP7, BoxB) into sgRNA loops to recruit coat proteins fused to FPs	[26, 27, 28] (Fig. 1B)	Flexible multicolor labeling, extensible with new aptamer-protein pairs	Background fluorescence increases with excess free reporters
Tandem FPs/Split FPs	Repetive	Fuse tandem FPs to dCas9 for stronger signals; split FPs reconstitute only at bound loci.	[25, 60] (Figs 1A and 2A)	Improves signal brightness; split-FP reduces nonspecific background	Limited amplification; increase protein size; reduce labeling efficiency
CRISPR-Sirius	Low-repetitive	Embed multiple optimized aptamers (8× MS2/PP7) for stable multicolor labeling	[33] (Fig. 2D)	Stronger signal via optimized aptamer scaffolds, stable long-term imaging	Still requires multiple sgRNAs and coat proteins
SunTag amplification	Repetitive	Fuse tandem GCN4 epitopes to dCas9, recruit scFv-FPs for strong amplification	[32, 61] (Fig. 2B)	24-fold stronger signals	Requires dCas9 engineering and expression of large scaffolds
Optogenetic SunTag (LEXY)	Repetitive	Combine SunTag with light-inducible nuclear export tag to remove unbound FPs	[62]	Improves SNR 2-2.5-fold by removing unbound FPs	Needs precise light control, not applicable in all systems
CRISPR-Tag/TriTag	Non-repetitive	Insert artificial CRISPR target arrays near loci, recruit multiple dCas9-FP complexes	[63, 64] (Fig. 2C)	Strong amplification at inserted cassettes, links DNA with RNA/protein imaging	Requires genome editing, not feasible in all cells/organisms
Tiled sgRNAs	Non-repetitive	Deliver large pools of sgRNAs via lentivirus to “paint” entire chromosome	[75] (Fig. 3A)	Enables whole-chromosome visualization; reveals conformational changes across cell cycle	Heavy sgRNA delivery burden; high complexity; limited practicality applications
Multi-sgRNA assembly	Non-repetitive	Multi-sgRNA assembly systems (20 + guides per plasmid; modular plasmid assembly)	[39, 40] (Fig. 3B)	Reduces delivery complexity; reproducible imaging of single-copy loci;	Difficult plasmid construction; moderate transfection burden
Cas12a arrays (CRISPR-delight)	Non-repetitive	Cas12a arrays process tandem crRNAs, enabling multiple guides from one transcript	[41] (Fig. 3C)	Compact design; efficient processing; fewer plasmids needed; scalable to multicolor imaging	Limited by Cas12a PAM constraints; Lack validations in diverse systems
LiveFISH/Oligo-LiveFISH/PRO-LiveFISH	Repetitive/non-repetitive	Deliver fluorescently labeled crRNA/dCas9 RNPs directly into cells	[42, 65, 76] (Figs 2F and 3D)	Rapid labeling without stable cell lines, high spatiotemporal resolution	Transient labeling, limited persistence
CRISPR-MB	Repetitive/non-repetitive	Hybridize fluorescent molecular beacons to engineered sgRNAs; FRET improves contrast	[66, 67] (Figs 2G and 4A)	Sensitive detection with only 2-3 sgRNAs, high contrast	Limited multiplexing, requires probe optimization
Quantum Dot (CRISPR-QD)	Non-repetitive	Tag dCas9 with peptides, conjugate streptavidin-QD probes for bright signals	[77] (Fig. 4B)	Bright, stable signals with minimal bleaching	Bulky probes, potential cytotoxicity
Casilio	Repetitive/non-repetitive	Engineer sgRNA with Pumilio binding sites to recruit programmable PUF-FPs	[35, 58] (Fig. 2E)	Programmable PUF domains expand multicolor labeling, reduced background	Complex sgRNA engineering, delivery burden
CRISPR-FISHer	Non-repetitive	Fuse FUS-IDR to dCas9-SunTag to induce local phase separation for signal clustering	[36] (Fig. 4C)	Single sgRNA imaging via phase separation, high sensitivity	Complex protein engineering, risk of condensate perturbation
SIMBA	Non-repetitive	Fuse HP1α to dCas9-SunTag, recruit GFP and induce heterochromatin condensation	[37]	Amplifies signal and modulates chromatin simultaneously	Couples imaging with epigenetic changes, which may confound results
CRISPR/Pepper-tDeg	Low-repetitive/non-repetitive	Insert Pepper RNA aptamers in sgRNA to recruit FP-tDeg fusions; fluorescence accumulates only at targets	[38, 78] (Fig. 4D)	Single sgRNA achieves bright, specific imaging with minimal background	Relatively new, generalizability across loci remains untested

## Engineering trade-offs and safety risks in CRISPR-Cas imaging

CRISPR-Cas9 has transformed live-cell genome imaging, but important technical and biological challenges remain. Here, we summarize the major imaging-related limitations and highlight emerging strategies to mitigate them. We also argue that these risks and trade-offs are still underappreciated in routine practice and should be treated as central design constraints rather than afterthoughts.

### SBR trade-offs and photobleaching constraints

A persistent challenge is the modest SBR. Fluorescently tagged dCas9 often produces diffuse nuclear fluorescence and causes nucleolar accumulation, complicating detection of true foci [20, 79]. Moreover, FRAP measurements indicate that dCas9 exhibits slow target turnover with multi-hour residence times in living cells, so bleached fluorophores are not rapidly replaced at bound loci, exacerbating signal loss during prolonged imaging [4, 31].

In multiplex imaging, SBR is often limited not only by probe design but also by stoichiometry among dCas effectors, sgRNAs, and recruited fluorescent binders [79, 80]. Transient plasmid transfection enables straightforward titration but can introduce strong cell-to-cell variability, whereas lentiviral delivery can improve stability yet still yield copy-number heterogeneity that inflates diffuse background. Practical solutions include promoter/induction tuning to minimize excess unbound components [79], consolidating modules into single polycistronic constructs (e.g. 2A/IRES) to enforce expression ratios, and, where feasible [80], single-copy genomic integration/targeted knock-in or BAC-based constructs for more uniform, near-physiological expression [63, 81]. While helpful for ratio control, polycistronic designs may impose expression constraints, and single-copy integration/knock-in or BAC-based approaches typically require more labor-intensive engineering and screening with reduced flexibility for rapid retargeting [80, 81]. Because nucleolar accumulation is frequently exacerbated by overexpression, maintaining the lowest effective expression levels is often the most direct way to improve SBR [79].

Strategies to improve SBR generally fall into two categories: (i) signal-boosting designs that increase fluorophore recruitment or brightness at bound loci (e.g. multimeric recruitment scaffolds such as SunTag [32, 61], tandem fluorescent proteins [25], multi-aptamer/scaffold recruitment [4, 33], Casilio [35, 58], phase separation-based signal clustering [36, 37], and bright or photostable probes such as quantum dots or fluorophore-labeled guides delivered as RNPs [42, 65, 76, 77]), and (ii) background-suppressing designs that restrict fluorescence to bound complexes or reduce unbound reporter pools (e.g. split-fluorescent protein complementation [59, 60], fluorogenic/conditional guide-probe designs such as CRISPR molecular beacons [66, 67], and degron-protected reporters such as CRISPR/Pepper-tDeg [38, 78]). Importantly, many SBR-boosting choices intersect with safety and side-effect considerations because increased expression burden and illumination requirements can exacerbate cellular stress and phototoxicity. We therefore use this section as a brief synthesis to motivate the safety issues discussed below.

### Replication interference and local genome instability

A dCas9 protein bound tightly to DNA can act as a physical roadblock to replication forks [44–46, 82]. *In vitro* experiments have demonstrated that dCas9-sgRNA complexes stall the progression of replication machinery from bacteriophage, bacterial, and even eukaryotic systems [44]. In living cells, this manifests as replication stress at the targeted locus. For instance, targeting a tandem repeat in yeast with dCas9 was shown to impede replication fork progression and induce focal genome instability, including copy-number variations in the repeat array [44–46].

Notably, even classical tagging methods can stall replication forks and trigger DNA damage responses, but CRISPR-Cas9 binding appears to cause an even stronger fork blockade accompanied by robust recruitment of DNA damage markers (e.g. γH2AX, 53BP1) [82]. Prolonged stalling of replication forks can lead to fork collapse or double-strand breaks, undermining genomic integrity [83]. To mitigate these issues, researchers may time imaging experiments to avoid replication of the tagged locus or use inducible systems to limit how long dCas9 is bound [46]. Ensuring that dCas9 is expressed at the lowest effective level and maybe releasing the block after imaging (e.g. via chemically or light-triggered dissociation) are potential strategies to reduce replication-associated damage, though such solutions remain experimental [84, 85]. We believe more research is needed to quantify the full extent of replication stress and DNA damage during imaging.

### Chromatin remodeling side-effects and transcriptional drift

dCas9 binding can alter chromatin state. Some studies report that dCas9 induces local chromatin opening, increasing accessibility and enhancing transcriptional responses to signaling cues [86]. Others find negligible changes in subnuclear positioning or adjacent gene expression [44]. These discrepancies suggest locus-specific effects depending on chromatin context. To minimize perturbation, guides should avoid promoters or regulatory regions and gene bodies as well, because dCas binding within transcribed regions can interfere with transcription and lead to repression [23, 75]. Going forward, we see a need for systematic, locus-by-locus mapping of chromatin accessibility and transcriptional consequences of dCas9 binding so that “safe harbor” windows for imaging can be defined empirically rather than assumed.

### Systemic cellular burden from sustained dCas9 expression

Unlike one-time editing, imaging requires sustained high levels of fluorescently tagged dCas9 and multiple sgRNAs, which can strain cellular systems (and may be further exacerbated in multiplex settings that co-express multiple Cas variants and fluorescent binders). Overexpression of dCas9 has been linked to growth defects in *Escherichia coli*, including abnormal morphology and widespread transcriptional dysregulation [48], and in human cells it can trigger TP53-dependent DNA damage responses leading to cell-cycle arrest [87]. Importantly, the observed “toxicity” in live-cell CRISPR imaging can arise from several non-exclusive sources, including (i) the burden of overexpressed proteins [88], (ii) prolonged DNA binding that may interfere with replication/transcription and induce replicative stress [22, 45], and (iii) phototoxicity during long-term time-lapse imaging [89]. Therefore, standardized toxicity metrics and a unified benchmarking workflow are crucial for comparing studies and guiding method optimization. If gRNAs target essential genes or accumulate off-targets, they may further reduce viability and potentially confound interpretations of chromatin dynamics [22].

To mitigate these effects, researchers can employ dosage control, using weaker or inducible promoters, or transient expression systems that restrict dCas9 to imaging windows [40]. Delivery of pre-assembled dCas9-sgRNA RNPs can also minimize long-term stress, though at the expense of persistence [42]. Exploring smaller Cas9 orthologs or engineered mini-Cas9 proteins offers another route to lower burden [90]. In practice, we recommend combining expression titration with conservative imaging settings and including basic controls/metrics of cellular stress to reduce imaging artifacts. Overall, careful titration of expression levels remains essential to balance signal detection with minimal cellular perturbation.

## Concluding remarks and future perspectives

CRISPR-based live-cell genome imaging has matured from a proof-of-concept into a versatile toolkit for exploring chromatin organization in real time [20, 21, 23]. A recent review by Park and Kim summarizes advances and outstanding challenges in CRISPR-Cas-based live genome imaging [74], whereas here we focus on engineering trade-offs and cellular constraints, particularly for imaging non-repetitive loci. The diverse strategies developed over the past decade, from multicolor orthogonal Cas9 systems [24, 25] to amplified single-locus tagging [35–38], collectively demonstrate how rapidly this field has advanced. These tools have already enabled unprecedented views of genome dynamics, illuminating processes ranging from chromatin compartment shifts in development to locus-specific movements during gene activation [41, 75]. What was once feasible only for repetitive telomeres or centromeres can now be achieved at many unique genomic sites, bringing us closer to routine visualization of virtually any locus of interest in living cells. However, routine biological adoption has lagged behind method development, and many recent quantitative studies of DNA locus dynamics continue to favor operator-array/FROS or ANCHOR-type genomic tagging systems [91–93], likely because robust CRISPR imaging at non-repetitive loci often requires extensive per-locus optimization and multi-component balancing to achieve sufficient SBR, with additional concerns about background, perturbation, and phototoxicity during long time-lapse imaging [74]. In contrast, genome-engineered operator-array/ANCHOR approaches typically provide stable expression over generations, enabling high-SBR, stable foci that once engineered cell lines are established [91–93].

Consistent with this progress, CRISPR-based live-cell genome imaging is increasingly enabling biological insights that directly link locus dynamics to nuclear function. By enabling direct, time-resolved tracking of defined loci, these approaches have linked locus mobility and repositioning to regulatory-state transitions, providing a dynamic layer beyond static 3D genome maps [63, 65, 76]. Improved labeling of non-repetitive loci has enabled quantitative interrogation of enhancer-promoter encounter dynamics and transcription factor binding at unique genomic sites and has facilitated linking locus motion to epigenetic and transcriptional states [39, 76, 94, 95]. At higher spatiotemporal resolution, single-copy tracking has supported quantitative descriptions of chromatin motion kinetics, including concepts such as “DNA communication time”—the typical time it takes for the motion of one DNA locus to become detectably correlated with the motion of a distant locus *in vivo* [42]—and simultaneous DNA-RNA-protein tracking is beginning to directly link locus motion to transcriptional output in single cells [52, 53].

Therefore, CRISPR-Cas imaging is still evolving, and several challenges remain as priorities for future work. One persistent issue is balancing signal brightness with background and cellular perturbation [30, 79]. Future designs will likely focus on “smart” signal amplification—e.g. labels or fluorescent probes that activate only when and where the target is bound, such as the CRISPR/Pepper-tDeg [38] and CRISPR MB [66, 67]. In parallel, compact effectors and reporters (engineered small/efficient dCas9 variants with minimal tags) offer a path to maintain binding fidelity while easing cellular burden [90, 96]. By reducing the size and dosage of the CRISPR imaging components, researchers aim to diminish replication fork interference, DNA damage responses, and general cytotoxicity while preserving robust visualization [44, 82]. We suggest that future generations of tools be explicitly benchmarked not only for imaging performance but also for a “perturbation budget,” quantifying how much they disturb replication, chromatin state, and cell physiology.

Another key step is improving delivery and control of CRISPR imaging reagents. High levels of fluorescent dCas9 and multiple sgRNAs work in immortalized lines but are poorly tolerated in sensitive or non-dividing cells [20, 21]. Transient and regulated systems—using inducible promoters or light-/drug-controlled dCas9 [40, 62, 97]—can enable “image on demand,” minimizing DNA occupancy. In parallel, pre-assembled RNP injection [42, 65, 76], viral vectors [23, 75], and nanoparticle carriers [98] provide short pulses of activity without continuous overexpression. Coupled with timing strategies, these approaches broaden compatibility to diverse cell types and whole organisms, and early work in plants and animals indicates that *in vivo* chromatin imaging is within reach [81, 99]. In our opinion, technologies that work robustly in primary, non-transformed, and *in vivo* systems—rather than only in a few permissive cell lines—should be prioritized as the next frontier for the field.

Looking ahead, the integration of CRISPR imaging with other modalities presents an exciting opportunity to deepen our understanding of nuclear functions. Recent demonstrations have shown that DNA loci can be tracked alongside their RNA transcripts (via dCas13) or even protein products [52, 53, 64, 65], pointing to a future where multi-faceted, real-time readouts of gene expression and 3D genome structure occur simultaneously in single cells. Extending this concept, one can envision combining CRISPR-Cas9 labeling with advanced microscopy techniques (such as super-resolution optics or high-speed lattice light-sheet imaging) to capture fine chromatin motions at millisecond temporal resolution or nanoscale spatial resolution [42, 100]. Additionally, as datasets from multi-locus tracking experiments grow, computational advances including machine learning-based image analysis will be crucial to extract meaningful patterns from the complex trajectories of chromatin in four dimensions [101].

In summary, CRISPR-Cas live-cell imaging stands as a transformative development in molecular cell biology, offering a level of flexibility and specificity in visualizing the genome that was previously unattainable. We anticipate that CRISPR-based imaging will not only refine our understanding of genome dynamics in fundamental research but also eventually inform biomedical insights by linking structural changes in the genome to functional outcomes in development and disease.

## Data Availability

This article is a review and does not generate new datasets.
